# Künstliche Intelligenz in der Mammadiagnostik: Hoffnungen und Herausforderungen

**DOI:** 10.1007/s00117-024-01409-7

**Published:** 2025-02-06

**Authors:** Matthias Dietzel, Alexandra Resch, Pascal A. T. Baltzer

**Affiliations:** 1 Department of Radiology, University Hospital Erlangen, Erlangen, Deutschland; 2 Department of Radiology, St. Francis Hospital Vienna, Sigmund Freud Private University Vienna, Vienna, Österreich; 3 Department of Biomedical Imaging and Image-Guided Therapy, Division of Molecular and Gender Imaging, Medical University of Vienna, Waehringer-Guertel 18–20, 1090 Vienna, Österreich

**Keywords:** Brustkrebs, Screening, Large Language Models, Doppelbefundung, Arzt-Patienten-Beziehung, Breast cancer, Screening, Large language models, Double diagnosis, Physician-patient relations

## Abstract

**Klinisches/methodisches Problem:**

Künstliche Intelligenz (KI) wird zunehmend im klinischen Alltag integriert. Vielen Anwendern ist der konkrete Nutzen noch unklar.

**Radiologische Standardverfahren:**

Prinzipiell stehen KI-Anwendungen für alle bildgebenden Verfahren zur Verfügung, wobei der Schwerpunkt in der Mammadiagnostik auf der Mammographie liegt.

**Methodische Innovationen:**

Künstliche Intelligenz verspricht eine Filterung von Untersuchungen in *negative* und *klar positive* Befunde und kann damit einen Teil der radiologischen Arbeitslast reduzieren. Andere Anwendungen sind noch nicht so weit etabliert.

**Leistungsfähigkeit:**

KI-Verfahren für die Mammographie und mit Einschränkungen auch die Tomosynthese erreichen bereits die Qualität radiologischer Befunder:innen.

**Bewertung:**

Bis auf Zweitmeinungsanwendungen/Triagierung in der Mammographie befinden sich die meisten Verfahren noch in der Entwicklung.

**Empfehlung für die Praxis:**

Derzeit müssen die meisten KI-Anwendungen durch potenzielle Anwender kritisch bezüglich ihrer Reife und ihres Benefits für die Praxis geprüft werden.

## Abstract (en)

**Clinical/methodical issue:**

Artificial intelligence (AI) is being increasingly integrated into clinical practice. However, the specific benefits are still unclear to many users.

**Standard radiological methods:**

In principle, AI applications are available for all imaging modalities, with a particular focus on mammography in breast diagnostics.

**Methodical innovations:**

AI promises to filter examinations into *negative* and *clearly positive* findings, and thereby reduces part of the radiological workload. Other applications are not yet as widely established.

**Performance:**

AI methods for mammography, and to a lesser extent tomosynthesis, have already reached the diagnostic quality of radiologists.

**Achievements:**

Except for second-opinion applications/triage in mammography, most methods are still under development.

**Practical recommendations:**

Currently, most AI applications must be critically evaluated by potential users regarding their maturity and practical benefits.

Die Mammadiagnostik gilt als ideales Anwendungsgebiet für künstliche Intelligenz (KI). Zum einen stehen durch das populationsbasierte Mammographie-Screening umfangreiche Bilddaten zur Verfügung, die in dieser Form einzigartig sind. Zudem wurden in nationalen Screening-Programmen umfassende Qualitätsstandards etabliert, und suspekte Befunde werden mit adäquaten Referenzstandards abgesichert. Damit sind zentrale Voraussetzungen für die Entwicklung eines leistungsfähigen KI-Systems gegeben. Zum anderen ist die praktische Notwendigkeit von KI in der Mammadiagnostik aufgrund der steigenden Arbeitsbelastung in diesem Fachgebiet offensichtlich.

Künstliche Intelligenz in der Mammadiagnostik ist eines der zentralen Diskussionsthemen in der senologischen Gemeinschaft. Aufgrund dieser allgegenwärtigen Präsenz wird es zunehmend schwieriger, zwischen tatsächlichen Möglichkeiten und unrealistischen Hypes zu differenzieren. Ziel dieses Artikels ist es daher, die realistischen Hoffnungen, die auf der KI in der Mammadiagnostik ruhen, zu beleuchten. Gleichzeitig stellen wir anhand ausgewählter Beispiele die großen Herausforderungen bei der Implementierung von KI in die klinische Praxis dar.

## Hoffnungen

### Welche klinischen Szenarien gibt es in der Mammadiagnostik?

Die Mamadiagnostik lässt sich grundsätzlich in 3 Szenarien einteilen: die Früherkennung, auch Screening genannt, die kurative Mammographie inklusive Assessment, und schließlich die Behandlungsführung von Patientinnen mit diagnostiziertem Mammakarzinom. Jedes dieser Szenarien unterscheidet sich klinisch erheblich voneinander, wodurch sich unterschiedliche Ansprüche und technische Lösungen für den Einsatz von KI ergeben. Daher ist es notwendig, die klinischen Rahmenbedingungen dieser Szenarien zunächst zu skizzieren:

#### Screening

Früherkennung gehört zur Sekundärprävention des Mammakarzinoms. Beim Screening werden klinisch inapparente Karzinome in scheinbar gesunden Frauen, auch Klientinnen genannt, entdeckt. Ziel ist es, die Morbidität und Mortalität des Mammakarzinoms in der Gesamtpopulation zu senken. Zudem können weniger invasive Operationen und weniger aggressive systemische Therapien erreicht werden.

In den meisten westlichen Industrieländern wird die Mammographie als Screening-Methode eingesetzt. Sie erfolgt dabei als Massenscreening. Ziel ist nicht, alle Karzinome zu finden, sondern unter gegebenen sozioökonomischen Rahmenbedingungen jene Fälle herauszufiltern, die eine gewisse (erhöhte) Wahrscheinlichkeit haben, dass ein Karzinom vorliegt und diese dann einer genaueren Abklärung zuzuführen (Assessment). Daher ist im Screening eine gewisse Rate an falsch-negativen Befunden unvermeidlich. Die Untersuchungen werden von geschultem technischem Personal durchgeführt und anschließend von Brustradiolog:innen mit speziellen Qualifikationen im Screening befundet [12].

#### Kurative Mammographie und Assessment

Kurative Mammographie meint die Untersuchung von symptomatischen Patientinnen, die sich etwa mit Schmerzen, Tastbefunden oder Ausfluss aus der Mamille vorstellen. Im Gegensatz dazu beschreibt das Assessment die weiterführende Untersuchung bei Screening-Recalls. Damit sind auffällige Befunde in Screening-Untersuchungen von klinisch gesunden Frauen gemeint. Obwohl Assessment und kurative Mammographie Unterschiede aufweisen, ist die diagnostische Aufgabenstellung für Radiolog:innen in beiden Fällen identisch. Daher werden die Begriffe in der radiologischen Literatur oft synonym verwendet. Sowohl im kurativen Setting wie auch im Assessment liegt der wesentliche Fokus auf dem sicheren Ausschluss eines Karzinoms. Ein hoher negativer Vorhersagewert ist dabei entscheidend, um das Vorliegen eines Tumors zuverlässig ausschließen zu können [4, 12].

#### Behandlungsführung

Unter senologischer „Behandlungsführung“ verstehen wir einen umfassenden Ansatz zur Planung, Überwachung und Anpassung der Behandlung von Brustkrebspatientinnen. Die Behandlungsführung beginnt mit der Entscheidung über die passende Therapieform, sei es Chemotherapie, Strahlentherapie oder Operation. Diese Entscheidung hängt von der Art des Tumors, dem Stadium der Krankheit und den individuellen Bedürfnissen der Patientin ab. Oft erfordert die Behandlungsführung die Zusammenarbeit eines multidisziplinären Teams, darunter Onkolog:innen, Chirurg:innen, Radiotherapeut:innen und Radiolog:innen. Das übergeordnete Ziel ist dabei, die bestmöglichen Behandlungsoptionen zu entwickeln und zu koordinieren. Während des gesamten Behandlungsverlaufs wird die Therapie regelmäßig überprüft und, wenn notwendig, an den Krankheitsverlauf und das Ansprechen der Patientin auf die Behandlung angepasst. Die Radiologie spielt in gesamten Prozess der *Behandlungsführung* eine zentrale Rolle. Dazu gehört zunächst das Staging, also die genaue Beurteilung der Tumorausdehnung. Dies umfasst nicht nur die bildgebende Diagnostik, sondern auch interventionelle Maßnahmen wie bildgestützte Biopsien und Herdmarkierungen. Ein weiterer essenzieller Aspekt in der Behandlungsführung ist das Ansprechen des Tumors auf die systemische Therapie [3].

### Welche KI-Anwendungen gibt es für diese klinische Szenarien?

#### Screening

**Abb. 1:**
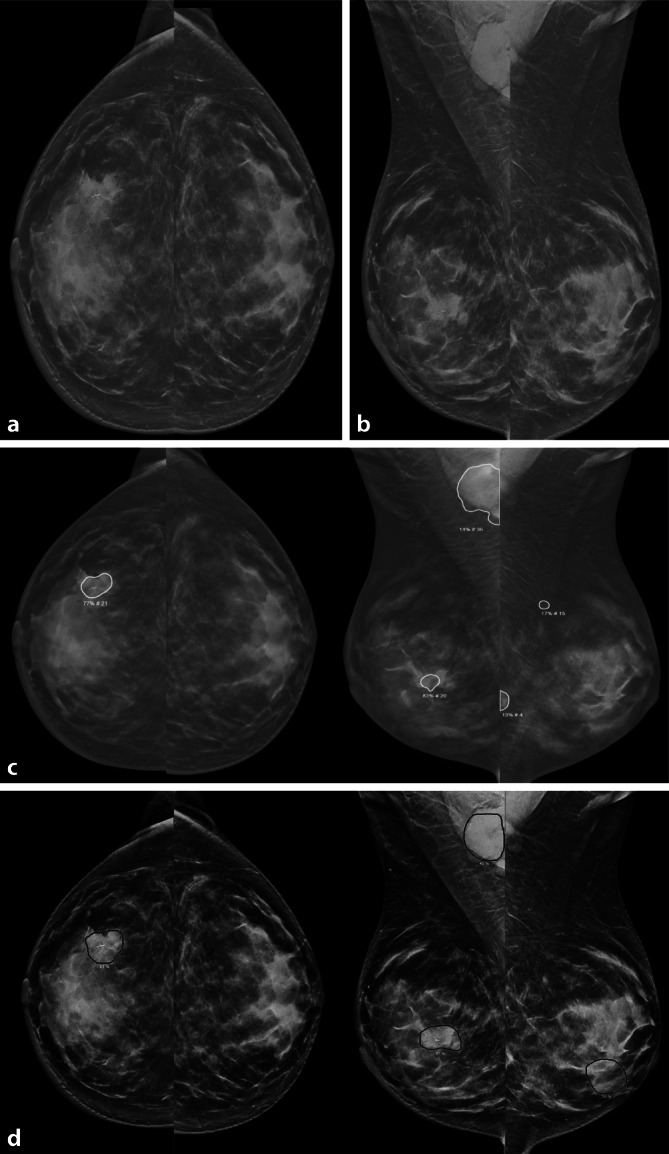
Korrekte Beurteilung eines Mammakarzinombefundes durch 2 KI-Systeme: Dargestellt ist die konventionelle Mammographie einer 75-jährigen Patientin in der kraniokaudalen (**a**, cc) und mediolateral-obliquen Projektion (**b**, mlo). Links auf 2 Uhr zeigt sich ein hyperdenser, irregulärer, unscharf begrenzter Herdbefund, der bereits histologisch als invasiv-lobuläres Karzinom gesichert und clipmarkiert wurde. In der mlo (**b**) ist zudem eine axilläre Lymphknotenmetastase sichtbar. Die Mammographie wurde unabhängig von 2 KI-Systemen analysiert (**c**, **d** jeweils cc und mlo). Beide detektierten sowohl den Indexherd als auch die Lymphknotenmetastase (*Markierung*). KI-System 1 (**c** iCAD-Score: 95) und KI-System 2 beurteilten übereinstimmend den Befund als hoch suspekt (Transpara-Score: 10). Beachte die kontralateralen falsch-positiven Befunde in **c** und **d**

Beim Mammographie-Screening ist in der Praxis mit 4 bis 6 entdeckten Mammakarzinome pro 1000 Untersuchungen zu rechnen. Das bedeutet, dass nur etwa in einer von 250 Untersuchungen ein Tumor diagnostiziert wird. Eine solche repetitive Analyse kann zur Ermüdung führen, wodurch die Gefahr besteht, dass genau jener eine Tumor übersehen wird. Um diese Risiken zu minimieren, ist im Massenscreening eine unabhängige Doppelbefundung gängige Praxis. Der damit verbundene hohe personelle Aufwand macht das Screening zu einem vielversprechenden Einsatzfeld für KI-Anwendungen. KI kann grundsätzlich zur Detektion von Befunden sowie zur Einschätzung der Malignomwahrscheinlichkeit eingesetzt werden (Abb. 1 und 2). Damit ist die Vorfilterung von Untersuchungen möglich, um klar negative Fälle auszusortieren und eindeutig positive Befunde automatisch an die Radiolog:in weiterzuleiten. Dieser Ansatz wird als Triage beschrieben und kann potenziell die Arbeitsbelastung verringern, die Fehlerquote reduzieren und den Bedarf an Fachpersonal senken. Perspektivisch könnte die KI sogar die Doppelbefundung ersetzen [7, 12].

**Abb. 2:**
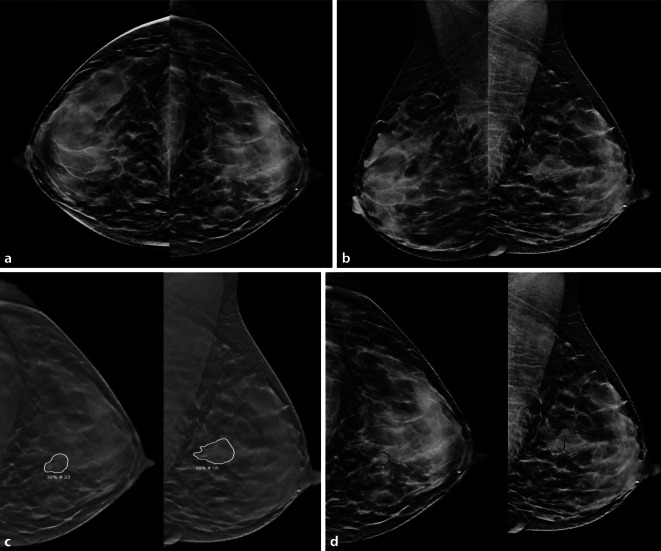
Widersprüchliche Beurteilung eines suspekten Befundes durch 2 KI-Systeme: Dargestellt ist die konventionelle Mammographie einer 74-jährigen Patientin in der kraniokaudalen (**a,** cc) und mediolateral-obliquen Projektion (**b**, mlo). Links retroareolär, an der dorsalen Parenchymgrenze, zeigt sich ein irregulärer, unscharf begrenzter isodenser Herdbefund, der konventionell als BI-RADS 4 und somit als abklärungsbedürftig eingestuft wurde. Die Mammographie wurde anschließend unabhängig von 2 KI-Systemen analysiert (**c**, **d** jeweils cc und mlo). Beide KI-Systeme detektierten den Befund (*Markierung*). KI-System 1 bewertete den Befund als *nicht suspekt* (iCAD-Score: 54). Im Gegensatz dazu stufte das zweite KI-System den Befund als *suspekt* ein (Transpara-Score: 10). Die histologische Verifizierung ergab Fibrose mit Adenose (B2) als finale Diagnose. Beachte einen weiteren falsch-positiven Befund in der mlo in **c**

Die derzeit besten wissenschaftlichen Daten zum Einsatz von KI in der Mammadiagnostik stammen aus dem Screening. Zwei wichtige Studien aus Schweden sollen hier exemplarisch erwähnt werden [9]. Die MASAI-Studie ist eine prospektive, randomisierte, kontrollierte Untersuchung an 80.033 Frauen. Sie zeigte, dass KI-unterstütztes Mammographie-Screening ähnliche Brustkrebsentdeckungsraten erreichte wie die konventionelle Doppelbefundung. Gleichzeitig sank die Arbeitsbelastung der Radiolog:innen um 44,3 % [9, 16]. Die prospektive ScreenTrustCAD-Studie untersuchte 55.581 Frauen und lieferte vergleichbare Ergebnisse. Der Austausch einer Radiolog:in durch KI führte hier sogar zu einer um 4 % höheren Krebsentdeckungsrate im Vergleich zur konventionellen Doppelbefundung [16]. Diese beiden in hochrangigen Fachzeitschriften veröffentlichten Studien unterstreichen das Potenzial der KI im Brustkrebsscreening. Sie bekräftigen die großen Hoffnungen, die auf der KI in der Mammadiagnostik ruhen.

Um das Potenzial der KI in der Mamadiagnostik differenziert zu verstehen, ist es aber wichtig zu betonen, dass diese Ergebnisse nur im Rahmen von populationsbezogenen Massenscreenings ihre Gültigkeit haben. In anderen Anwendungsfeldern der Mamadiagnostik sind unterschiedliche Resultate zu erwarten. Selbst innerhalb des Mammographiescreenings gibt es erhebliche Unterschiede. Während in Deutschland das klassische Massenscreening etabliert ist, wird in Österreich das Brustkrebsscreening als individualisiertes Programm durchgeführt. Hier sieht der Radiologe jede Patientin einzeln, was ganz andere Anforderungen an die Integration der KI in den Workflow stellt.

#### Kurative Mammographie und Assessment

Der sichere Tumorausschluss ist das Ziel sowohl in der kurativen Situation als auch im Assessment. Um diese Sicherheit zu gewährleisten, führen Radiolog:innen weiterführende Bildgebung und/oder bildgeführte Biopsien durch. Der dafür notwendige personelle und apparative Aufwand ist hoch und belastet das Gesundheitssystem. Gleichzeitig entsteht durch dieses Vorgehen eine erhebliche Anzahl falsch-positiver Befunde. KI kann hier die Mammadiagnostik unterstützen und so eine sicheren Tumorausschluss ermöglichen. Dabei greifen KI-Systeme nicht nur auf radiologische Bilddaten zurück. Sie integrieren auch klinische Informationen der Patientin – wie Alter, Risikostatus – und berücksichtigen den zeitlichen Verlauf aller Befunde. Dadurch wird eine objektivere Diagnosefindung unterstützt, die Genauigkeit erhöht, und es können Empfehlungen für weiterführende Diagnostik gegeben werden [4, 7].

#### Behandlungsführung

Der komplexe Prozess der Behandlungsführung bei Mammakarzinomen erfordert ein hohes Maß an Expertise, multidisziplinäre Kommunikation sowie eine effiziente Informationsübermittlung. Dabei ist der Einsatz multimodaler Bildgebung, minimal-invasiver Biopsien und Tumormarkierungen durch die Radiologie von zentraler Bedeutung. Der Arbeitsaufwand ist dabei hoch, nur eingeschränkt standardisierbar und bleibt individuell variabel. Obwohl gerade solche Situationen für die KI hohes Potenzial bieten, steht die Entwicklung KI-basierter Entscheidungshilfen aufgrund der Notwendigkeit ausreichend großer und balancierter Trainingsdatensätze vor größeren Herausforderungen als im Screening oder der diagnostischen Abklärung [3, 7].

Ein typisches Anwendungsszenario in der Behandlungsplanung ist die Beurteilung des Ansprechens eines Karzinoms auf eine Therapie mittels KI-gestützter radiologischer Verfahren. Diese ermöglichen eine objektivere und präzisere Bewertung der Therapieansprache. Darüber hinaus ist auch eine prätherapeutische Prognose des Ansprechens auf eine bestimmte Therapie grundsätzlich möglich. Obwohl diesen Verfahren auf wissenschaftlichen Tagungen und in der Fachliteratur viel Beachtung geschenkt wird, hat die KI-gestützte Beurteilung des Therapieansprechens in der klinischen Praxis derzeit keinen wertschöpfenden Einfluss. Aus den gewonnenen Informationen leiten sich in den aktuellen Leitlinien oder Fachempfehlungen keine klaren Managemententscheidungen ab. Grund dafür ist, dass derzeit weder eine Deeskalation noch eine Eskalation der medikamentösen Therapie im Falle eines besonders guten oder schlechten Ansprechens vorgesehen ist, ebenso wenig eine zeitliche Anpassung der Therapie [3, 8].

Ein weiteres potenzielles Anwendungsgebiet von KI ist die Vorhersage des molekularen Subtyps. Aktuell erfolgt die Bestimmung des molekularen Subtyps noch über standardisierte Surrogatmarker. Dabei spielen der Hormonrezeptorstatus, die HER2/neu-Amplifikation sowie die proliferative Aktivität des Tumors, gemessen durch die Ki67-Färbung eine zentrale Rolle. Auf Basis dieser molekularen Charakterisierung lassen sich spezifische therapeutische Maßnahmen ableiten. Aggressive Tumorsubtypen werden dabei gemäß den aktuellen Leitlinien meist neoadjuvant systemisch behandelt. KI-gestützte radiologische Verfahren sind in der Lage, den molekularen Subtyp eines Tumors vorhersagen zu können. Ein praxisrelevanter Vorteil dieser Technologie liegt darin, dass die Informationen im Rahmen eines *One-Stop-Shop-*Ansatzes zeiteffizient gekoppelt mit der diagnostischen Bildgebung bereitgestellt werden können. Die KI-basierte Analyse umfasst zusätzlich den gesamten Tumor und ist nicht, wie bei einer konventionellen Gewebeuntersuchung, auf kleine, bioptisch gesicherte Areale beschränkt. Dadurch verspricht die KI-gestützte Analyse eine umfassendere, präzisere und zeiteffizientere Tumorcharakterisierung. Auch diese Überlegungen unterstreichen das Potenzial der KI für die Behandlungsführung von Patientinnen mit Mammakarzinom [10].

### Welche KI-Anwendungen gibt es noch?

Neben der eigentlichen Verarbeitung von Bilddaten, wie wir sie im vorherigen Kapitel beschrieben haben, gibt es eine Vielzahl weiterer KI-Anwendungen in der Mammadiagnostik. Exemplarisch möchten wir im Folgenden Large Language Models (LLMs) genauer beleuchten.

LLMs bieten vielversprechende Einsatzmöglichkeiten in der Mammadiagnostik bei der Erstellung, Überprüfung und Kommunikation von Befunden [1, 5]. LLMs repräsentieren KI-Systeme, die in der Lage sind, natürliche Sprache zu verstehen, Texte zu erzeugen und Inkonsistenzen in solchen zu erkennen. In der Mammadiagnostik können LLMs daher die Befundungszeit erheblich beschleunigen und somit Radiolog:innen entlasten. Darüber hinaus unterstützen sie Radiolog:innen bei der automatisierten Erstellung von Fließtexten, basierend auf wenigen Angaben. LLMs helfen effektiv, Befunde zu überprüfen und in der klinischen Praxis Befundfehler zu erkennen. So können Seitenverwechslungen vermieden und Diskrepanzen zwischen Befundtext und Zusammenfassung entdeckt werden. Damit können LLMs sowohl für die interne als auch externe Qualitätskontrolle eingesetzt werden. Obwohl LLMs noch eine junge KI-Anwendung in der Radiologie darstellen, haben sie enormes Potenzial. Dieses ist aus unserer Sicht mindestens so hoch zu werten wie die Verbesserung der diagnostischen Aufgaben selbst, die wir im vorherigen Kapitel besprochen haben [1, 5].

## Herausforderungen

### Kann KI zur Wertschöpfung beitragen?

Eine wesentliche Motivation für den Einsatz von KI in der Mammadiagnostik ist es, das Dilemma der immer größer werdenden Untersuchungszahlen bei einer begrenzten Anzahl von Radiolog:innen zu lösen. Damit ist bereits das Konzept der „value-based medicine“ umschrieben. Deren Grundgedanke lässt sich wie folgt präzisieren: Neue Technologien müssen nicht nur in experimentellen wissenschaftlichen Studien funktionieren, sondern auch im klinischen Alltag einen messbaren Nutzen („value“) generieren. Trotz der erheblichen Fortschritte in der KI-Forschung der letzten Jahre scheint das wertschöpfende Potenzial der KI in der Mammadiagnostik nach wie vor am wenigsten untersucht zu sein [14, 17].

Die meisten KI-Lösungen in der Mammadiagnostik konzentrieren sich auf die Bildinterpretation. In diesem Bereich sind aktuelle KI-Systeme oft gleichwertig mit menschlichen Untersuchern. Doch auch wenn dieses Ergebnis per se beeindruckend ist, bleibt die Frage offen, ob dadurch so bereits ein tatsächlicher Mehrwert („value“) vorliegt [7, 20].

Selbst dort, wo es plausibel erscheint, dass KI zur Wertschöpfung beitragen könnte, lohnt sich ein genauerer Blick. Eine vieldiskutierte Anwendung von KI im Screening ist die Triage, das Aussortieren von sicher gutartigen Untersuchungen. Expert:innen argumentieren, dass durch Triage die Arbeitsbelastung der Befunder:innen im Screening sinken wird. Dieses auf den ersten Blick überzeugende Narrativ hält jedoch einer genaueren Betrachtung nicht stand. Der Arbeitsaufwand bei der Bildinterpretation ist ungleich verteilt. Eine Brust mit fettreichem Gewebe ist deutlich schneller zu beurteilen als eine Brust mit dichtem Gewebe. Sortiert man die *einfachen* Fälle aus, sinkt zwar die absolute Anzahl der Befunde, gleichzeitig verdichtet sich die Arbeit auf schwierige und zeitaufwändige Fälle. Daneben kann die Triage das Rating der Befunder:innen beeinflussen; dieser Effekt kann im weiteren Sinn als „calibration bias“ beschrieben werden [2]. Diese Überlegungen verdeutlichen, dass die Frage, ob KI tatsächlich zur Wertschöpfung in der Mammadiagnostik beiträgt, noch nicht beantwortet ist.

### Kann KI das Vertrauen der Patientinnen gewinnen?

KI-Systeme, sind nicht fehlerfrei und müssen daher fester Bestandteil des Fehlermanagements sein. Die Grundlage jeglichen ärztlichen Handelns ist eine tragfähige Beziehung zwischen Patient:in und Ärzt:in. Nur durch dieses Vertrauen lässt sich ein professioneller, zielgerichteter und gleichzeitig empathischer Umgang mit Fehlern ermöglichen. Dies stellt besondere Herausforderungen dar, da die Integration von KI eine klare Verantwortungsteilung sowie ein tiefes Verständnis der möglichen Fehlerquellen erfordert. Nur so wird es uns gelingen, das Vertrauen unserer Patientinnen in die KI zu stärken und so den erfolgreichen Einsatz in der klinischen Praxis zu erreichen [6, 19].

Bei menschlich verursachten Fehlern ermöglicht der persönliche Austausch eine direkte Übernahme von Verantwortung. Dies ist in schwierigen Situationen essenziell, um das Vertrauen der Patientin zu erhalten. Bei KI-bedingten Fehlbefunden fehlt jedoch diese persönliche Komponente. Das wird in der Praxis die Beziehung zwischen Patientin und Ärzt:in belasten und das Fehlermanagement erschweren. Gerade weil KI-Fehler aus menschlicher Sicht mitunter nicht nachvollziehbar sind, erschweren diese den Umgang mit solchen Situationen. Ebenso bekannt ist, dass im selben Befund verschiedene KI-Systeme unterschiedliche Ergebnisse liefern können, wie in Abb. 2 demonstriert wird [15]. Dies kann das Vertrauen der Patient:in in die Technologie beeinträchtigen, insbesondere wenn Fehlbefunde, wie etwa falsch-negative Diagnosen trotz offensichtlicher maligner Läsionen, auftreten. Solche Fälle sind in der Literatur gut dokumentiert (vgl. z. B. Abb. 4b in [13]).

Daher bleibt – unabhängig von der allgemeinen diagnostischen Genauigkeit der KI – der Vertrauensaspekt entscheidend, um auch in seltenen Fällen von KI-bedingten Fehlbefunden aufzuarbeiten und eine optimale Fehlerkultur zu etablieren. Umfragen zur Einstellung von Frauen gegenüber KI-gestütztem Screening bestätigen diese Einschätzung. Die Befragten betonten, dass das Vertrauensverhältnis zur Ärzt:in entscheidend für die Akzeptanz und den Erfolg der KI in der Mammadiagnostik ist [11].

Patientinnenvertreter:innen könnten eine zentrale Rolle dabei spielen, das Vertrauen der Patientinnen in die neue Technik zu entwickeln. Sie könnten effektiv die Bedenken der Patientinnen an die Ärzt:innen weitergeben und gleichzeitig über die Möglichkeiten und Chancen der KI-Technologie aufklären. So könnten Patientinnenvertreter:innen sowohl die Perspektive der Patientinnen vertreten als auch deren Informierung in ihrer eigenen Gruppe maßgeblich fördern.

### Können KI-Hersteller objektive Forschung durchführen?

Abschließend möchten wir ein zentrales, jedoch wenig beachtetes Problem der KI in der Mammadiagnostik ansprechen. Softwarehersteller nehmen zunehmend, ähnlich wie die Pharmaindustrie in der Onkologie, erheblichen Einfluss auf die Gestaltung und Durchführung von KI-Studien. Dies hat nachweisbare Auswirkungen auf die wissenschaftlichen Ergebnisse:

Zunächst hängt die Qualität von KI-Tools entscheidend von der Qualität der Trainingsdaten ab. KI-Hersteller kaufen häufig Daten von Bildgebungszentren ein, die in der Regel nicht universitär sind und deren Annotationen sowie Referenzstandards intransparent bleiben. In so einem Szenario können wichtige Kriterien einer qualitätskontrollierten unabhängigen Forschung nicht erreicht werden. Das an diesen Daten entwickelte KI-Tool wird zunächst intern getestet und anschließend in Zusammenarbeit mit einer universitären Einrichtung in einer offiziellen Studie validiert. Auch in diesem Stadium ist die Forschung nicht unabhängig von KI-Herstellern. Vielmehr finden sich regelhaft unter den Autor:innen solcher Studien Angestellte des Unternehmens, das die jeweilige Software vertreibt [13, 18].

Diese gängige Praxis in der KI-Szene widerspricht in Teilen Qualitätskriterien konventioneller radiologischer Forschung. Es gibt kaum einen Unterschied dazu, wenn ein Hersteller eines Klinikinformationssystems sein Softwareupdate anhand eigener Kriterien testet und eine selektive Verbesserung als wissenschaftlichen Fortschritt vermarktet. Im Grunde handelt es sich hier um Werbung unter dem Deckmantel wissenschaftlicher Legitimation. Wissenschaftlich weniger versierte Anwender:innen sehen das Qualitätssiegel einer Studie und sollen dadurch zum Kauf des Produkts bewegt werden.

Die Einflussnahme der KI-Hersteller auf Studienergebnisse ist in einer rezenten hochrangig publizierten Metaanalyse wissenschaftlich belegt [20]. Hier wurde die diagnostische Genauigkeit von KI-Systemen mit der von Radiolog:innen verglichen. Es wurden sowohl Studien verglichen, die von unabhängigen wissenschaftlichen Institutionen durchgeführt wurden, als auch solche, bei denen ein KI-Hersteller direkt involviert war.

Zunächst war die diagnostische Genauigkeit von KI-Systemen dabei unabhängig davon, ob ein Hersteller an der Studie beteiligt war oder nicht. Dieses Ergebnis ist plausibel und unterstreicht die Objektivität der KI-Diagnose.

Nicht plausibel ist das zweite Ergebnis der Studie: Demnach war die diagnostische Genauigkeit von Radiolog:innen signifikant schlechter in solchen Studie, in denen KI-Hersteller involviert war. Dies hat zur Folge, dass die Vorteile der KI in solchen Studien signifikant größer ausfielen als in solchen Arbeiten, in denen nur unabhängig Institution beteiligt waren. Die Bewertung von KI-Systemen ist demnach nachweislich von KI-Herstellern geprägt [20].

Als radiologische Gemeinschaft müssen wir uns der zunehmenden Einflussnahme der KI-Industrie bewusstwerden und aktiv nach Lösungen suchen, um dieser Entwicklung entgegenzuwirken. Dieser Prozess beginnt mit dem Bewusstsein für die Einflussnahme KI-Industrie auf die radiologische Forschung. Zu diesem Zweck haben wir in Tab. 1 relevante Kriterien für objektive Forschung zusammengefasst. Diese können bei der Lektüre von Fachliteratur herangezogen werden, um die Qualität der KI-Literatur unabhängig zu beurteilen.

**Tab. 1:** Kriterien für eine transparente KI-Forschung

Kriterium	Erläuterung
Offenlegung von Daten	Zusammensetzung, Herkunft und Qualität der Trainingsdaten sollten vollständig offengelegt werden
Zugänglichkeit und Kontrolle der Trainingsdaten	Trainingsdaten müssen öffentlich zugänglich und unabhängig überprüfbar sein
Verpflichtende Testung an einem Repository	Hersteller müssen ihre KI-Software an einem einheitlichen Repository testen, das für das spezifische Setting relevant ist
Kommunikation von KI-Schwachstellen	Spezifische Schwachstellen der KI sollten eindeutig identifiziert und kommuniziert werden
Unabhängigkeit von Studien	Strenge Maßstäbe für die Unabhängigkeit von Studien, besonders bei Industriekollaborationen, einhalten

*KI* künstliche Intelligenz

Diese Tabelle fasst essenzielle Kriterien für eine transparente KI-Forschung in der Mammadiagnostik zusammen. Diese können die kritische Lektüre von Fachliteratur unterstützten

## Fazit für die Praxis

- Die Anwendung von künstlicher Intelligenz (KI) weckt große Hoffnungen, zentrale Herausforderungen in der Mammadiagnostik zu lösen.
- In naher Zukunft erwarten wir den größten Effekt durch den Ersatz der Zweitbefundung im Screening durch KI.
- Neben der reinen diagnostischen Tätigkeit bietet KI auch deutliche Vorteile in der Befundkommunikation und bei der Qualitätskontrolle.
- Die KI in der Mammadiagnostik steht vor erheblichen Herausforderungen, wie z. B. Dokumentation des tatsächlichen Nutzens gemäß der „value-based medicine“ sowie die Stärkung des Vertrauens der Patientinnen in diese Technologie.
- Eine große Herausforderung ist auch die enge Verbindung zwischen Industrie und akademischer Radiologie und die hierdurch bedingte Verzerrung von Studienergebnissen.
- Um dieser Problematik zu begegnen, ist die radiologische Gemeinschaft gefordert, sich dieser Einflussnahme bewusst zu werden und die Kompetenz zu entwickeln, solche Verflechtungen zu erkennen und zu verhindern – nur so kann eine erfolgreiche und unabhängige Implementierung der KI in die Mammadiagnostik zum Wohl der Patientinnen in der Zukunft gelingen.
